# Disease Mechanisms and Therapeutic Approaches in *C9orf72* ALS-FTD

**DOI:** 10.3390/biomedicines9060601

**Published:** 2021-05-25

**Authors:** Keith Mayl, Christopher E. Shaw, Youn-Bok Lee

**Affiliations:** 1 Department of Basic and Clinical Neuroscience, Institute of Psychiatry, Psychology and Neuroscience, Maurice Wohl Clinical Neuroscience Institute, King’s College London, 5 Cutcombe Road, London SE5 9RT, UK; keith.mayl@kcl.ac.uk (K.M.); chris.shaw@kcl.ac.uk (C.E.S.); 2 UK Dementia Research Institute, King’s College London, 5 Cutcombe Road, London SE5 9RT, UK; 3 King’s MND Care and Research Centre, Academic Neurosciences Centre, King’s College Hospital, London SE5 9RS, UK

**Keywords:** ALS, FTD, *C9orf72*, C9ALS-FTD, RNA, therapies

## Abstract

A hexanucleotide repeat expansion mutation in the first intron of *C9orf72* is the most common known genetic cause of amyotrophic lateral sclerosis and frontotemporal dementia. Since the discovery in 2011, numerous pathogenic mechanisms, including both loss and gain of function, have been proposed. The body of work overall suggests that toxic gain of function arising from bidirectionally transcribed repeat RNA is likely to be the primary driver of disease. In this review, we outline the key pathogenic mechanisms that have been proposed to date and discuss some of the novel therapeutic approaches currently in development.

## 1. Background

Amyotrophic lateral sclerosis (ALS) is a devastating, progressive, neurodegenerative disease characterised by the loss of upper and lower motor neurons [1]. It typically presents in the fifth or sixth decade of life and is characterised by profound muscle wasting, relentless weakness of limb and/or bulbar muscles leading to paralysis, and death secondary to respiratory compromise within 3–5 years from symptom onset [1]. Frontotemporal dementia (FTD) is a heterogeneous, progressive, neurodegenerative disorder encompassing a broad range of distinct clinical phenotypes associated with changes in executive function, behaviour, language, and motor dysfunction [2]. In the vast majority of both ALS and FTD cases, the aetiology is unknown and is said to be sporadic. However, in a subset of cases, ranging up to 10% for ALS [1] and 30% for FTD [3], the disease is familial and is linked to a specific genetic mutation. To date, there is no effective disease-modifying treatment available for ALS or FTD, and clinical management is based on alleviating the symptoms and disability that come with the diseases. In 2006, a locus on chromosome 9 was linked to ALS and FTD, and this was further validated in a large genome-wide association study in 2011 [4,5]. These findings paved the way to the landmark discovery that an intronic hexanucleotide GGGGCC (G4C2) repeat expansion in the chromosome 9 open reading frame 72 (*C9orf72*) gene is the most common cause of both familial ALS and FTD, and cemented the clinical, genetic, and molecular link between the two diseases, giving rise to the *C9orf72*-associated ALS-FTD (C9ALS-FTD) disease spectrum [6,7].

Clinically, C9ALS-FTD is generally indistinguishable from what is considered to be classic ALS or behavioural variant-FTD (bvFTD) [2]. However, even within these categories there is extensive heterogeneity amongst individual cases, and it is not uncommon for diverse clinical presentations to occur within the same family. Cognitive impairment occurs far more commonly in C9ALS than in cases not related to the expansion mutation [8], and the occurrence of ALS and FTD within the same patient is recognised. Some studies have shown an increase in bulbar-onset ALS in the presence of the *C9orf72* expansion mutation [9,10], though this is not corroborated across all studies. Hallucinations and delusions are far more common in C9FTD cases than in other forms of bvFTD [11,12], and patients may even be diagnosed with schizophrenia when these features are particularly prominent [13]. Overall, expansions within this gene are responsible for up to 20–40% of familial FTD and ALS cases, respectively, as well as 10% of apparently sporadic ALS cases in people of European ancestry [14]. The expansion mutation has also been occasionally identified in people diagnosed with Parkinson’s disease [15], multiple system atrophy [16], and corticobasal syndrome [17].

**Figure 1:**
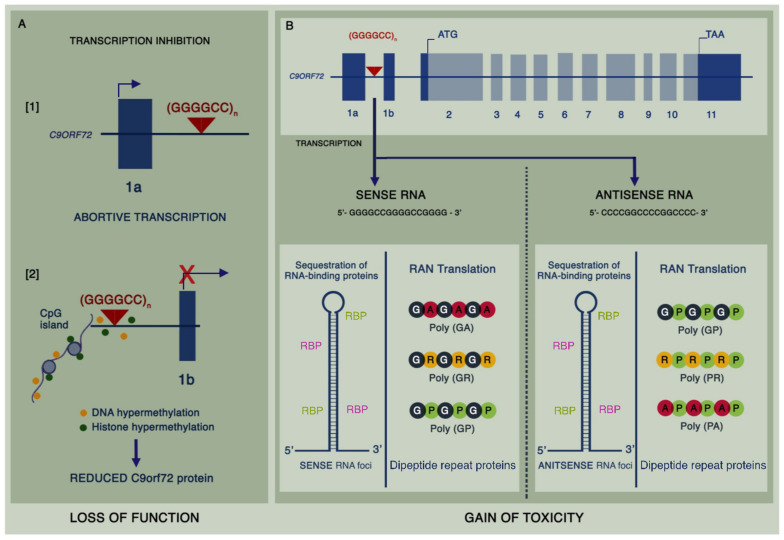
Pathological mechanisms associated with the hexanucleotide repeat expansion in C9ALS-FTD: (**A**) The presence of the expanded GGGGCC mutation in intron 1 of *C9orf72* potentially causes abortive transcription from exon 1a leading to haploinsufficiency of C9orf72 protein (1). Loss of function is further compounded by reduced transcription secondary to hypermethylation of both DNA and histones (2). (**B**) Bidirectionally transcribed repeat RNA is proposed to be toxic by sequestering RNA-binding proteins into RNA foci, and through the formation of aberrant dipeptide repeat proteins arising from repeat-associated non-AUG-dependent (RAN) translation.

In the years that have followed the discovery of the mutation, significant progress has been made in unearthing the mechanisms thought to be responsible for neurodegeneration in C9ALS-FTD. Most cases are due to G4C2 repeat expansions reaching several hundreds to thousands of repeats in size [18]. The vast majority of healthy individuals have less than 10 repeats, though an intermediate repeat size of 24–30 repeats may confer increased risk [19], and reports of disease with as few as 30 repeats have been described [20]. Three major hypotheses have emerged as being mechanistic (Figure 1), namely: loss of function due to haploinsufficiency of the C9orf72 protein, a toxic gain of function from intranuclear sense and antisense RNA foci arising from bidirectional transcription of the expansion mutation, and toxic gain of function from dipeptide repeat (DPR) proteins formed by repeat-associated non-ATG-dependent (RAN) translation [21]. The exact contribution of each of these mechanisms to disease is still unclear, and there may be a combined effect, in conjunction with other downstream processes, leading to neurotoxicity.

A pathological hallmark for the vast majority of ALS cases, and approximately half of all FTD cases, is the presence of Transactive Response DNA-Binding Protein 43 (TDP-43) inclusions within neuronal and glial cells [22]. The role of TDP-43 in neurodegeneration has been extensively investigated and is reviewed elsewhere [23,24]. C9ALS-FTD belongs to this group of TDP-43 proteinopathies. However, in C9ALS-FTD cases, the hallmark pathology is the presence of p62-positive (and TDP-43-negative) cytoplasmic and intranuclear inclusions, which were subsequently shown to colocalise with DPR protein aggregates [25]. Nonetheless, this overlap of C9ALS-FTD with other forms of the disease may reflect a common point of convergence across the disease spectrum [26].

## 2. Disease Mechanisms

### 2.1. Loss-of-Function Mechanisms

It has previously been shown that C9orf72 protein levels are decreased, giving rise to haploinsufficiency in C9ALS-FTD [27]. *C9orf72* messenger RNA (mRNA) is alternatively spliced such that at least five different transcripts are generated, of which only two retain the G4C2 expansion mutation [28]. Interestingly, whilst overall mature *C9orf72* mRNA is decreased, the levels of sense and antisense transcripts containing intron 1, and hence the expansion mutation, are increased in C9ALS-FTD [29]. C9orf72 is structurally related to the differentially expressed in normal and neoplastic cells (DENN) guanine nucleotide exchange factor (GEF) proteins, which mediate activation of Rab proteins [30]. Knockdown of *C9orf72* in human cell lines has been shown to inhibit autophagy and leads to cytoplasmic aggregation of TDP-43 and the accumulation of p62 [31]. Similarly in *C9orf72* knockout mouse models, an accumulation of autophagy substrates including p62 has been observed [32]. These findings have established a role for C9orf72 as a modulator of autophagy, and it is thought that this process is primarily driven by its interaction with the Unc-51 like kinase-1 (ULK1) complex [33]. Furthermore, C9orf72 has also been implicated in endolysosomal trafficking, and studies in C9ALS-FTD human iPSC-derived motor neurons have shown there is a reduction in lysosomes corresponding to reduced vesicular trafficking [34].

Several *C9orf72* knockout mouse models have been generated to study loss-of-function mechanisms in C9ALS-FTD. Most models have been linked to autoimmune dysregulation whereby knockout mice develop a fatal phenotype characterised by cervical lymphadenopathy, splenomegaly, and increased levels of inflammatory cytokines [35,36,37]. Transcriptomic analysis in human samples has shown that *C9orf72* transcripts are highly expressed in CD14^+^ myeloid cells, which are involved in innate and adaptive immunity, further suggesting an important role for C9orf72 in regulating the immune response [38]. However, none of the knockout models to date develop a neurodegenerative phenotype in keeping with C9ALS-FTD, suggesting that a loss-of-function mechanism alone is not sufficient to cause disease.

### 2.2. Gain-of-Function Mechanisms

The presence of intranuclear RNA foci derived from sense and antisense *C9orf72* transcripts in C9ALS-FTD is well-recognised. Repeat RNA foci have been observed in the motor cortex, frontal cortex, hippocampus, cerebellum, spinal motor neurons, interneurons, and glial cells of patients with C9ALS-FTD [39]. Toxicity from RNA foci is thought to be driven by the sequestration of RNA-binding proteins. Interactome analyses have shown that heterogeneous nuclear ribonucleoproteins (hnRNPs) are particularly prone to bind to *C9orf72* repeat RNA. Isoforms of hnRNP-H, in particular, are capable of binding to repeat RNA and may lead to neurotoxicity by disrupting alternative splicing of intron–exon cassettes [40]. Furthermore, the RNA recognition motif of hnRNPs is shared with other proteins implicated in ALS pathogenesis including TDP-43 and fused in sarcoma (FUS) [41]. Interactions are mediated by the underlying structure of mutant RNA including hairpins, G-quadruplexes, and R-loops (DNA–RNA heteroduplexes), and thus, the structural variation within G4C2 repeats may in turn affect several RNA pathways leading to a multi-hit gain-of-function model [42].

Despite the intronic location of the expansion mutation, sense and antisense repeat RNA is exported to the cytoplasm and is translated in every reading frame to form five different DPR proteins: poly(GP), poly(GA), poly(GR) from the sense strand, and poly(PR), poly(PA), and poly(GP) from the antisense strand [29]. Numerous studies support the toxicity of DPR proteins. It has previously been shown that arginine-containing DPRs drive neurodegeneration in a *Drosophila* eye model [43]. Preventing RAN translation by interrupting the repeats with stop codons in each reading frame within this model subsequently rescued the phenotype. In another study, expression of poly(GA) proteins in the chick and mouse CNS lead to neurodegeneration [44], and behavioural deficits linked to the ability of poly(GA) to aggregate and sequester various proteins, including those involved in nucleocytoplasmic transport and proteasomal protein degradation [45]. In human cell lines, the arginine-rich poly(PR) and poly(GR) were shown to be particularly toxic by binding to nucleoli and disrupting RNA processing when exogenously applied to cultured astrocytes [46]. Furthermore, poly(GR) and poly(PR) have been shown to cause TDP-43 mislocalisation through a mechanism by which accumulation of DPRs leads to enhanced karyopherin dysfunction and, thus, impaired nuclear TDP-43 import [47]. Several other downstream mechanisms have been implicated in C9ALS-FTD, including dysfunctional nucleocytoplasmic transport, impaired assembly of membrane-less organelles such as stress granules and nucleoli through the effect of arginine-containing DPRs on liquid–liquid phase separation [48,49], and translational inhibition by binding of poly(GR) and poly(PR) to mRNA [50]. Translational inhibition and altered stress granules are indeed implicated in ALS and FTD cases associated with FUS and TDP-43 inclusions and may represent a common mechanism across the spectrum [51]. Overall, several in vitro and in vivo models support the toxicity of the arginine-rich DPRs across multiple pathways in C9ALS-FTD, with some evidence supporting toxicity of poly(GA).

## 3. Therapeutic Approaches in C9ALS-FTD

**Figure 2:**
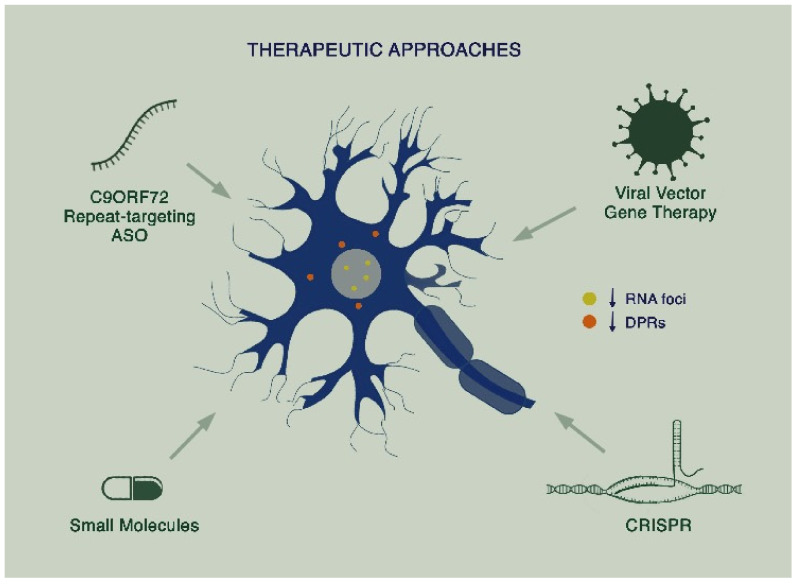
Proposed therapeutic approaches in C9ALS-FTD: Targeting of toxicity arising from sense and antisense RNA foci and dipeptide repeat proteins (DPRs) through a combination of therapies including repeat-targeting antisense oligonucleotides (ASOs), viral vector gene therapy, small molecules, and CRISPR.

As knowledge of disease mechanisms in C9ALS-FTD has grown, therapies specifically targeting the mutation have started to emerge. To date, these have largely focused on alleviating the toxic gain of function arising from the G4C2 repeat expansion mutation (Figure 2).

### 3.1. Targeting C9orf72 Repeat RNA or DNA

#### 3.1.1. Antisense Oligonucleotides

The most clinically advanced therapeutic approaches currently involve the use of antisense oligonucleotides (ASOs). ASOs are short, synthetic, single-stranded DNA molecules designed to bind to mRNA by complementary base pairing. This leads to the degradation of mRNA via RNase H-mediated decay, providing a strategy to knockdown toxic mRNA and thus protein. This approach is under investigation to treat ALS secondary to mutations in the superoxide dismutase 1 (*SOD1*) gene, with initial results confirming a reduction in CSF SOD1 protein following intrathecal ASO (BIIB067/tofersen) administration in patients [52]. A global phase 3 clinical trial is currently underway to determine the clinical efficacy of this approach (ClinicalTrials.gov identifier: NCT02623699). ASOs can also be used to modulate RNA splicing events to favour the formation of particular RNA species, thereby augmenting the production of a gene product. This approach has been used to successfully treat spinal muscular atrophy in children [53,54].

ASOs targeting the *C9orf72* repeat expansion mutation have shown encouraging results in various animal models of disease and in human cells. Rescue of disease-specific pathologies such as RNA foci, dipeptide repeats (DPRs), and defective nucleocytoplasmic transport (NCT) have been observed in *C9orf72* patient fibroblasts and iPSC-derived neurons following treatment with ASOs [55,56,57]. Similar approaches also alleviate neurodegeneration in *Drosophila* [58] and reduce RNA foci and DPRs in transgenic mouse models [35]. Of note, selective degradation of repeat RNA is achievable without complete knockdown of C9orf72 protein. This is an important consideration given the potential synergistic effect of haploinsufficiency in disease pathogenesis [59,60] and the immune dysregulation observed in *C9orf72* knockout mouse models [32,35,36]. A phase 1 clinical trial to determine the safety and tolerability of an ASO (BIIB078) targeting the sense strand of *C9orf72* transcripts containing the repeat expansion is currently underway in patients with C9-ALS (ClinicalTrials.gov identifier: NCT03626012). Furthermore, an ASO (ION363/Jacifusen) targeting *fused in sarcoma* (*FUS*) mRNA is currently under investigation in an expanded access program at Columbia University for patients with ALS secondary to mutations in *FUS*.

#### 3.1.2. RNA Interference

Alternative approaches to gene silencing have made use of the RNA interference (RNAi) pathway. Short non-coding RNA molecules, such as small interfering RNA (siRNA) and artificial microRNA (miRNA), have been proposed as therapeutic strategies in C9ALS-FTD. Single-stranded siRNA (ss-siRNA) targeting the hexanucleotide repeat have been shown to bind to mutant *C9orf72* transcripts leading to a reduction in RNA foci in patient-derived fibroblasts in an RNAi-dependent and independent manner [61]. Targeting of the transcription elongation factor SPT4 with siRNA reduces levels of sense and antisense *C9orf72* repeat transcripts, thereby decreasing RNA foci and DPRs in *C9orf72* iPSC-neurons and mitigating neurodegeneration in a *Drosophila* model of disease [62].

More recently, an artificial microRNA (miRNA) strategy incorporating adeno-associated virus (AAV) has been proposed as a gene therapy to target C9ALS-FTD. In this study, miRNA designed to target intron 1 of *C9orf72* was shown to reduce mutant RNA transcripts in iPSC neurons derived from C9FTD patients following transduction with AAV5. This resulted in selective knockdown of mutant transcripts, while preserving normal *C9orf72* mRNA levels [63]. Bilateral intra-striatal injection of AAV5 expressing miRNA targeting total *C9orf72* mRNA in a *C9orf72* transgenic mouse model led to efficient transduction of the cortex, striatum, and midbrain. This resulted in a 20–40% reduction in total and mutant *C9orf72* mRNA and ~20% reduction in the number of frontal cortical neurons containing sense RNA foci in treated mice [63]. A similar viral vector strategy incorporating AAV encoding miRNA targeting *SOD1* mRNA has been tested in an experimental human study involving two patients with *SOD1* ALS [64]. A phase 1 clinical trial is currently underway for Huntington Disease in which intrastriatally delivered AAV5 encoding artificial miRNA targeting Huntington mRNA is under investigation (ClinicalTrials.gov Identifier: NCT04120493). These ventures represent a new era of AAV-based gene therapies with the potential to transform treatment of neurological diseases.

#### 3.1.3. CRISPR-Cas 9

Advances in gene editing with CRISPR/Cas technology have made it possible to target the hexanucleotide repeat expansion at the genomic level. CRISPR/Cas (clustered regularly interspaced short palindromic repeats/CRISPR-associated protein) is an adaptation of a naturally occurring genome editing process that occurs in bacteria. Studies have shown that a CRISPR–Cas9 system targeting either the hexanucleotide repeat DNA [65], or repeat RNA [66] reduces RNA foci and DPR levels in cell lines.

#### 3.1.4. Small Molecules

An alternative strategy is to use small molecules which target the secondary structure of *C9orf72* repeat RNA. TMPyP4 binds *C9orf72* repeat RNA in vitro and has been shown to reduce sequestration of RBPs [67]. In another study, TMPyP4 was shown to rescue nucleocytoplasmic transport defects and neurodegeneration in a *Drosophila* model overexpressing hexanucleotide repeats [58]. Small molecules targeting the G-quadruplex have also been shown to reduce the production of RNA foci and DPRs when applied to patient-derived iPSC neurons [68]. More recently, peptidylic inhibitors that bind to G4C2 repeat RNA have been proposed as another therapeutic strategy in C9ALS-FTD by alleviating nucleolar stress in vitro and in vivo [69].

Collectively, multiple studies have shown that targeting of mutant RNA or DNA is a viable therapeutic approach in C9ALS-FTD. At present ASOs are the most clinically advanced candidates, though several other emerging therapies, as outlined above, hold promise.

### 3.2. Targeting Dipeptide Repeats

There is a large body of work suggesting DPRs arising from RAN translation of *C9orf72* repeat RNA are toxic (extensively reviewed elsewhere [21,70]). This has sparked interest in developing strategies which directly target DPRs. Clearance of toxic proteins via passive or active immunological approaches is a strategy that has been pursued in other neurodegenerative disorders such as Alzheimer’s disease and Parkinson disease. Passive immunisation against DPRs using targeted antibodies has been proposed as a therapeutic strategy for C9ALS-FTD. Poly(GA)-specific antibodies have been shown to reduce intracellular poly(GA) aggregation and the seeding activity of C9ALS-FTD brain extracts [71]. More recently, another study showed that poly(GA)-specific antibodies improved behaviour, decreased neuroinflammation, and increased survival in a *C9orf72* transgenic mouse model [72]. In this study, peripherally delivered antibodies were able to cross the blood–brain barrier to enter cells and target DPR aggregates, suggesting this could be a potentially viable strategy in humans. Clearance of DPRs by other mechanisms has also been explored. Overexpression of the small heat shock protein HSPB8 has been shown to reduce DPR levels, possibly via the autophagy pathway [73].

### 3.3. Targeting RAN Translation

RAN translation is a recognised pathological phenomenon in several repeat expansion disorders, such as spinocerebellar ataxia type 8 [74] and myotonic dystrophy type 1 [75]. Manipulation of RAN translation therefore represents a potential therapeutic approach for multiple diseases. A recent study has identified RNA-dependent protein K (PKR) as a regulator of RAN translation [76]. Metformin (an insulin sensitiser commonly used in the treatment of diabetes mellitus) was shown to inhibit PKR phosphorylation leading to a reduction in RAN proteins in vitro and in vivo, mitigating disease in a C9ALS-FTD transgenic mouse model [76]. A phase 2 clinical trial exploring the safety and therapeutic potential of metformin in C9ALS-FTD is underway (ClinicalTrials.gov Identifier: NCT04220021).

### 3.4. Targeting Downstream Mechanisms

Alternative strategies with therapeutic potential have targeted the downstream mechanisms implicated in C9ALS-FTD, such as nucleocytoplasmic transport (NCT) and stress granule formation. Reducing nuclear export by targeting the nuclear export factors, serine/arginine-rich splicing factor 1 (SRSF1) or exportin 1, alleviates toxicity in *Drosophila* models of C9ALS-FTD [58,77]. Aside from genetic knockdown approaches used to reduced SRSF1 and exportin 1, small molecules called selective inhibitors of nuclear export (SINE) were also used and shown to be effective [58]. A phase 1 clinical trial involving the use of a SINE molecule in ALS patients is currently underway (ClinicalTrials.gov Identifier: NCT03945279). The inhibition of stress granule formation using ASOs targeting ataxin 2 has been shown to ameliorate NCT dysfunction and neurodegeneration in iPSC-neurons derived from patients with C9ALS and in vivo [58]. Reduction in ataxin 2 has subsequently been shown to significantly extend survival in a TDP-43 transgenic mouse model of ALS, generating great interest in this approach [78].

### 3.5. Limitations to Therapies

Therapies have largely focused on knocking down toxicity arising from putative gain-of-function mechanisms. However, there is increasing evidence that loss-of-function mechanisms may contribute to disease in synergy with gain-of-function mechanisms [60]. Aside from toxicity arising from DPRs and RNA foci, haploinsufficiency of C9orf72 protein is a recognised event in disease. This may be the result of the expansion mutation undergoing hypermethylation in an attempt to silence gene expression [79]. A picture is now beginning to emerge, whereby both toxic gain-of-function and loss-of-function mechanisms are likely to be involved in disease pathogenesis.

It is clear that knockdown strategies, such as those described above, should aim to avoid total loss of C9orf72 protein given the deleterious effects seen in mouse models. This implies that knockdown approaches ought to be selective such that only mutant RNA is targeted. In this way, non-mutant transcripts would be preserved, thereby avoiding further C9orf72 haploinsufficiency. Furthermore, it may be desirable to increase C9orf72 protein levels, while simultaneously knocking down toxic RNA species or DPRs. Such an approach may be critical to restore normal C9orf72 levels whilst alleviating the toxicity arising from repeat RNA. A further factor to consider in C9ALS-FTD is that toxicity is thought to arise from both sense and antisense strands, and thus, separate approaches may be required to target both.

As ASOs are currently the most clinically advanced therapeutic candidates, it is important to note that intrathecal delivery of these molecules requires patients to undergo repeated lumbar punctures. Although lumbar puncture is generally a safe procedure, it is not without complications, and adverse events relating to the procedure have been frequently reported in clinical trials of ASOs [52]. The procedure also requires skilled medical professionals to safely complete, and may limit widespread availability of these therapies, particularly in resource-deficient healthcare systems. Non-ASO-based approaches, such as viral vector gene therapies as described above, may overcome some of the limitations of ASO therapies, as a single administration may be sufficient to achieve sustained therapeutic effects. However, the medical expertise and resources required to deliver such therapies should not be underestimated. Development of novel modes of delivery may be required to achieve widespread transduction within the CNS [80].

## 4. Conclusions

Since the discovery of the G4C2 repeat expansion in intron 1 of *C9orf72*, collective efforts within the community have identified key mechanistic disease processes. This has translated in numerous therapeutic approaches, of which several are already in clinical development. It is clear that treatment of ALS-FTD, be it familial or sporadic, is likely to require multiple interventions, targeting numerous aspects of disease, potentially including both gain-of-function and loss-of-function mechanisms. The emergence of novel therapeutics in the field in the form of ASOs, viral vector gene therapies, CRISPR/Cas-9 systems, or small molecules holds promise and may change our understanding and management of neurodegenerative disorders at large.

## Data Availability

The study did not report new data.

## References

[1] Brown R.H., Al-Chalabi A. (2017). Amyotrophic Lateral Sclerosis. N. Engl. J. Med..

[2] Rohrer J.D., Isaacs A.M., Mizlienska S., Mead S., Lashley T., Wray S., Sidle K., Fratta P., Orrell R.W., Hardy J. (2015). C9orf72 Expansions in Frontotemporal Dementia and Amyotrophic Lateral Sclerosis. Lancet Neurol..

[3] Rohrer J.D., Guerreiro R., Vandrovcova J., Uphill J., Reiman D., Beck J., Isaacs A.M., Authier A., Ferrari R., Fox N.C. (2009). The Heritability and Genetics of Frontotemporal Lobar Degeneration. Neurology.

[4] Vance C., Al-Chalabi A., Ruddy D., Smith B.N., Hu X., Sreedharan J., Siddique T., Schelhaas H.J., Kusters B., Troost D. (2006). Familial Amyotrophic Lateral Sclerosis with Frontotemporal Dementia Is Linked to a Locus on Chromosome 9p13.2-21.3. Brain.

[5] Shatunov A., Mok K., Newhouse S., Weale M.E., Smith B., Vance C., Johnson L., Veldink J.H., van Es M.A., van den Berg L.H. (2010). Chromosome 9p21 in Sporadic Amyotrophic Lateral Sclerosis in the UK and Seven Other Countries: A Genome-Wide Association Study. Lancet Neurol..

[6] DeJesus-Hernandez M., Mackenzie I.R., Boeve B.F., Boxer A.L., Baker M., Rutherford N.J., Nicholson A.M., Finch N.C.A., Flynn H., Adamson J. (2011). Expanded GGGGCC Hexanucleotide Repeat in Noncoding Region of C9ORF72 Causes Chromosome 9p-Linked FTD and ALS. Neuron.

[7] Renton A.E., Majounie E., Waite A., Simón-Sánchez J., Rollinson S., Gibbs J.R., Schymick J.C., Laaksovirta H., van Swieten J.C., Myllykangas L. (2011). A Hexanucleotide Repeat Expansion in C9ORF72 Is the Cause of Chromosome 9p21-Linked ALS-FTD. Neuron.

[8] Millecamps S., Boillée S., Le Ber I., Seilhean D., Teyssou E., Giraudeau M., Moigneu C., Vandenberghe N., Danel-Brunaud V., Corcia P. (2012). Phenotype Difference between ALS Patients with Expanded Repeats in C9ORF72 and Patients with Mutations in Other ALS-Related Genes. J. Med. Genet..

[9] Cooper-Knock J., Hewitt C., Highley J.R., Brockington A., Milano A., Man S., Martindale J., Hartley J., Walsh T., Gelsthorpe C. (2012). Clinico-Pathological Features in Amyotrophic Lateral Sclerosis with Expansions in C9ORF72. Brain.

[10] Byrne S., Elamin M., Bede P., Shatunov A., Walsh C., Corr B., Heverin M., Jordan N., Kenna K., Lynch C. (2012). Cognitive and Clinical Characteristics of Patients with Amyotrophic Lateral Sclerosis Carrying a C9orf72 Repeat Expansion: A Population-Based Cohort Study. Lancet Neurol..

[11] Snowden J.S., Rollinson S., Thompson J.C., Harris J.M., Stopford C.L., Richardson A.M.T., Jones M., Gerhard A., Davidson Y.S., Robinson A. (2012). Distinct Clinical and Pathological Characteristics of Frontotemporal Dementia Associated with C9ORF72 Mutations. Brain.

[12] Mahoney C.J., Beck J., Rohrer J.D., Lashley T., Mok K., Shakespeare T., Yeatman T., Warrington E.K., Schott J.M., Fox N.C. (2012). Frontotemporal Dementia with the C9ORF72 Hexanucleotide Repeat Expansion: Clinical, Neuroanatomical and Neuropathological Features. Brain.

[13] Galimberti D., Reif A., Dell’Osso B., Kittel-Schneider S., Leonhard C., Herr A., Palazzo C., Villa C., Fenoglio C., Serpente M. (2014). The C9ORF72 Hexanucleotide Repeat Expansion Is a Rare Cause of Schizophrenia. Neurobiol. Aging.

[14] Scotter E.L., Chen H.J., Shaw C.E. (2015). TDP-43 Proteinopathy and ALS: Insights into Disease Mechanisms and Therapeutic Targets. Neurotherapeutics.

[15] Lesage S., Le Ber I., Condroyer C., Broussolle E., Gabelle A., Thobois S., Pasquier F., Mondon K., Dion P.A., Rochefort D. (2013). C9orf72 Repeat Expansions Are a Rare Genetic Cause of Parkinsonism. Brain.

[16] Goldman J.S., Quinzii C., Dunning-Broadbent J., Waters C., Mitsumoto H., Brannagan T.H., Cosentino S., Huey E.D., Nagy P., Kuo S.H. (2014). Multiple System Atrophy and Amyotrophic Lateral Sclerosis in a Family with Hexanucleotiderepeat Expansions in C9orf72. JAMA Neurol..

[17] Lindquist S.G., Duno M., Batbayli M., Puschmann A., Braendgaard H., Mardosiene S., Svenstrup K., Pinborg L.H., Vestergaard K., Hjermind L.E. (2013). Corticobasal and Ataxia Syndromes Widen the Spectrum of C9ORF72 Hexanucleotide Expansion Disease. Clin. Genet..

[18] van Blitterswijk M., DeJesus-Hernandez M., Niemantsverdriet E., Murray M.E., Heckman M.G., Diehl N.N., Brown P.H., Baker M.C., Finch N.C.A., Bauer P.O. (2013). Association between Repeat Sizes and Clinical and Pathological Characteristics in Carriers of C9ORF72 Repeat Expansions (Xpansize-72): A Cross-Sectional Cohort Study. Lancet Neurol..

[19] van der Zee J., Gijselinck I., Dillen L., Van Langenhove T., Theuns J., Engelborghs S., Philtjens S., Vandenbulcke M., Sleegers K., Sieben A. (2013). A Pan-European Study of the C9orf72 Repeat Associated with FTLD: Geographic Prevalence, Genomic Instability, and Intermediate Repeats. Hum. Mutat..

[20] Iacoangeli A., Al Khleifat A., Jones A.R., Sproviero W., Shatunov A., Opie-Martin S., Morrison K.E., Shaw P.J., Shaw C.E., Fogh I. (2019). C9orf72 Intermediate Expansions of 24–30 Repeats Are Associated with ALS. Acta Neuropathol. Commun..

[21] Balendra R., Isaacs A.M. (2018). C9orf72-Mediated ALS and FTD: Multiple Pathways to Disease. Nat. Rev. Neurol..

[22] Neumann M., Sampathu D.M., Kwong L.K., Truax A.C., Micsenyi M.C., Chou T.T., Bruce J., Schuck T., Clark C.M., McCluskey L.F. (2006). Ubiquitinated TDP-43 in Frontotemporal Lobar Degeneration and Amyotrophic Lateral Sclerosis. Science.

[23] Cohen T.J., Lee V.M.Y., Trojanowski J.Q. (2011). TDP-43 Functions and Pathogenic Mechanisms Implicated in TDP-43 Proteinopathies. Trends Mol. Med..

[24] De Boer E.M.J., Orie V.K., Williams T., Baker M.R., De Oliveira H.M., Polvikoski T., Silsby M., Menon P., Van Den Bos M., Halliday G.M. (2021). TDP-43 Proteinopathies: A New Wave of Neurodegenerative Diseases. J. Neurol. Neurosurg. Psychiatry.

[25] Al-Sarraj S., King A., Troakes C., Smith B., Maekawa S., Bodi I., Rogelj B., Al-Chalabi A., Hortobágyi T., Shaw C.E. (2011). P62 Positive, TDP-43 Negative, Neuronal Cytoplasmic and Intranuclear Inclusions in the Cerebellum and Hippocampus Define the Pathology of C9orf72-Linked FTLD and MND/ALS. Acta Neuropathol..

[26] Ling S.C., Polymenidou M., Cleveland D.W. (2013). Converging Mechanisms in Als and FTD: Disrupted RNA and Protein Homeostasis. Neuron.

[27] Waite A.J., Bäumer D., East S., Neal J., Morris H.R., Ansorge O., Blake D.J. (2014). Reduced C9orf72 Protein Levels in Frontal Cortex of Amyotrophic Lateral Sclerosis and Frontotemporal Degeneration Brain with the C9ORF72 Hexanucleotide Repeat Expansion. Neurobiol. Aging.

[28] Douglas A.G.L. (2018). Non-Coding RNA in C9orf72-Related Amyotrophic Lateral Sclerosis and Frontotemporal Dementia: A Perfect Storm of Dysfunction. Non Coding RNA Res..

[29] Mori K., Weng S., Arzberger T., May S., Rentzsch K., Van Broeckhoven C., Haass C., Edbauer D. (2013). The C9orf72 GGGGCC Repeat Is Translated into Aggregating Dipeptide-Repeat Proteins in FTLD/ALS. Science.

[30] Levine T.P., Daniels R.D., Gatta A.T., Wong L.H., Hayes M.J. (2013). The Product of C9orf72, a Gene Strongly Implicated in Neurodegeneration, Is Structurally Related to DENN Rab-GEFs. Bioinformatics.

[31] Sellier C., Campanari M., Julie Corbier C., Gaucherot A., Kolb-Cheynel I., Oulad-Abdelghani M., Ruffenach F., Page A., Ciura S., Kabashi E. (2016). Loss of C9 ORF 72 Impairs Autophagy and Synergizes with PolyQ Ataxin-2 to Induce Motor Neuron Dysfunction and Cell Death. EMBO J..

[32] O’Rourke J.G., Bogdanik L., Yáñez A., Lall D., Wolf A.J., Muhammad A.K.M.G., Ho R., Carmona S., Vit J.P., Zarrow J. (2016). C9orf72 Is Required for Proper Macrophage and Microglial Function in Mice. Science.

[33] Webster C.P., Smith E.F., Bauer C.S., Moller A., Hautbergue G.M., Ferraiuolo L., Myszczynska M.A., Higginbottom A., Walsh M.J., Whitworth A.J. (2016). The C9orf72 Protein Interacts with Rab1a and the ULK 1 Complex to Regulate Initiation of Autophagy. EMBO J..

[34] Shi Y., Lin S., Staats K.A., Li Y., Chang W.H., Hung S.T., Hendricks E., Linares G.R., Wang Y., Son E.Y. (2018). Haploinsufficiency Leads to Neurodegeneration in C9ORF72 ALS/FTD Human Induced Motor Neurons. Nat. Med..

[35] Jiang J., Zhu Q., Gendron T.F., Saberi S., McAlonis-Downes M., Seelman A., Stauffer J.E., Jafar-nejad P., Drenner K., Schulte D. (2016). Gain of Toxicity from ALS/FTD-Linked Repeat Expansions in C9ORF72 Is Alleviated by Antisense Oligonucleotides Targeting GGGGCC-Containing RNAs. Neuron.

[36] Burberry A., Suzuki N., Wang J.Y., Moccia R., Mordes D.A., Stewart M.H., Suzuki-Uematsu S., Ghosh S., Singh A., Merkle F.T. (2016). Loss-of-Function Mutations in the C9ORF72 Mouse Ortholog Cause Fatal Autoimmune Disease. Sci. Transl. Med..

[37] Atanasio A., Decman V., White D., Ramos M., Ikiz B., Lee H.C., Siao C.J., Brydges S., Larosa E., Bai Y. (2016). C9orf72 Ablation Causes Immune Dysregulation Characterized by Leukocyte Expansion, Autoantibody Production, and Glomerulonephropathy in Mice. Sci. Rep..

[38] Rizzu P., Blauwendraat C., Heetveld S., Lynes E.M., Castillo-Lizardo M., Dhingra A., Pyz E., Hobert M., Synofzik M., Simón-Sánchez J. (2016). C9orf72 Is Differentially Expressed in the Central Nervous System and Myeloid Cells and Consistently Reduced in C9orf72, MAPT and GRN Mutation Carriers. Acta Neuropathol. Commun..

[39] Mizielinska S., Lashley T., Norona F.E., Clayton E.L., Ridler C.E., Fratta P., Isaacs A.M. (2013). C9orf72 Frontotemporal Lobar Degeneration Is Characterised by Frequent Neuronal Sense and Antisense RNA Foci. Acta Neuropathol..

[40] Lee Y.B., Chen H.J., Peres J.N., Gomez-Deza J., Attig J., Štalekar M., Troakes C., Nishimura A.L., Scotter E.L., Vance C. (2013). Hexanucleotide Repeats in ALS/FTD Form Length-Dependent RNA Foci, Sequester RNA Binding Proteins, and Are Neurotoxic. Cell Rep..

[41] Prudencio M., Belzil V.V., Batra R., Ross C.A., Gendron T.F., Pregent L.J., Murray M.E., Overstreet K.K., Piazza-Johnston A.E., Desaro P. (2015). Distinct Brain Transcriptome Profiles in C9orf72-Associated and Sporadic ALS. Nat. Neurosci..

[42] Haeusler A.R., Donnelly C.J., Rothstein J.D. (2016). The Expanding Biology of the C9orf72 Nucleotide Repeat Expansion in Neurodegenerative Disease. Nat. Rev. Neurosci..

[43] Mizielinska S., Grönke S., Niccoli T., Ridler C.E., Clayton E.L., Devoy A., Moens T., Norona F.E., Woollacott I.O.C., Pietrzyk J. (2014). C9orf72 Repeat Expansions Cause Neurodegeneration in Drosophila through Arginine-Rich Proteins. Science.

[44] Lee Y.B., Baskaran P., Gomez-Deza J., Chen H.J., Nishimura A.L., Smith B.N., Troakes C., Adachi Y., Stepto A., Petrucelli L. (2017). C9orf72 Poly GA RAN-Translated Protein Plays a Key Role in Amyotrophic Lateral Sclerosis via Aggregation and Toxicity. Hum. Mol. Genet..

[45] Zhang Y.J., Gendron T.F., Grima J.C., Sasaguri H., Jansen-West K., Xu Y.F., Katzman R.B., Gass J., Murray M.E., Shinohara M. (2016). C9ORF72 Poly(GA) Aggregates Sequester and Impair HR23 and Nucleocytoplasmic Transport Proteins. Nat. Neurosci..

[46] Kwon I., Xiang S., Kato M., Wu L., Theodoropoulos P., Wang T., Kim J., Yun J., Xie Y., McKnight S.L. (2014). Poly-Dipeptides Encoded by the C9orf72 Repeats Bind Nucleoli, Impede RNA Biogenesis, and Kill Cells. Science.

[47] Solomon D.A., Stepto A., Au W.H., Adachi Y., Diaper D.C., Hall R., Rekhi A., Boudi A., Tziortzouda P., Lee Y.B. (2018). A Feedback Loop between Dipeptide-Repeat Protein, TDP-43 and Karyopherin-α Mediates C9orf72-Related Neurodegeneration. Brain.

[48] Boeynaems S., Bogaert E., Kovacs D., Konijnenberg A., Timmerman E., Volkov A., Guharoy M., De Decker M., Jaspers T., Ryan V.H. (2017). Phase Separation of C9orf72 Dipeptide Repeats Perturbs Stress Granule Dynamics. Mol. Cell.

[49] Lee K.H., Zhang P., Kim H.J., Mitrea D.M., Sarkar M., Freibaum B.D., Cika J., Coughlin M., Messing J., Molliex A. (2016). C9orf72 Dipeptide Repeats Impair the Assembly, Dynamics, and Function of Membrane-Less Organelles. Cell.

[50] Kanekura K., Yagi T., Cammack A.J., Mahadevan J., Kuroda M., Harms M.B., Miller T.M., Urano F. (2016). Poly-Dipeptides Encoded by the C9ORF72 Repeats Block Global Protein Translation. Hum. Mol. Genet..

[51] Bowden H.A., Dormann D. (2016). Altered MRNP Granule Dynamics in FTLD Pathogenesis. J. Neurochem..

[52] Miller T., Cudkowicz M., Shaw P.J., Andersen P.M., Atassi N., Bucelli R.C., Genge A., Glass J., Ladha S., Ludolph A.L. (2020). Phase 1-2 Trial of Antisense Oligonucleotide Tofersen for SOD1 ALS. N. Engl. J. Med..

[53] Finkel R.S., Mercuri E., Darras B.T., Connolly A.M., Kuntz N.L., Kirschner J., Chiriboga C.A., Saito K., Servais L., Tizzano E. (2017). Nusinersen versus Sham Control in Infantile-Onset Spinal Muscular Atrophy. N. Engl. J. Med..

[54] Mercuri E., Darras B.T., Chiriboga C.A., Day J.W., Campbell C., Connolly A.M., Iannaccone S.T., Kirschner J., Kuntz N.L., Saito K. (2018). Nusinersen versus Sham Control in Later-Onset Spinal Muscular Atrophy. N. Engl. J. Med..

[55] Donnelly C.J., Zhang P.W., Pham J.T., Heusler A.R., Mistry N.A., Vidensky S., Daley E.L., Poth E.M., Hoover B., Fines D.M. (2013). RNA Toxicity from the ALS/FTD C9ORF72 Expansion Is Mitigated by Antisense Intervention. Neuron.

[56] Lagier-Tourenne C., Baughn M., Rigo F., Sun S., Liu P., Li H.R., Jiang J., Watt A.T., Chun S., Katz M. (2013). Targeted Degradation of Sense and Antisense C9orf72 RNA Foci as Therapy for ALS and Frontotemporal Degeneration. Proc. Natl. Acad. Sci. USA.

[57] Gendron T.F., Chew J., Stankowski J.N., Hayes L.R., Zhang Y.J., Prudencio M., Carlomagno Y., Daughrity L.M., Jansen-West K., Perkerson E.A. (2017). Poly(GP) Proteins Are a Useful Pharmacodynamic Marker for C9ORF72-Associated Amyotrophic Lateral Sclerosis. Sci. Transl. Med..

[58] Zhang K., Donnelly C.J., Haeusler A.R., Grima J.C., Machamer J.B., Steinwald P., Daley E.L., Miller S.J., Cunningham K.M., Vidensky S. (2015). The C9orf72 Repeat Expansion Disrupts Nucleocytoplasmic Transport. Nature.

[59] Shao Q., Liang C., Chang Q., Zhang W., Yang M., Chen J.F. (2019). C9orf72 Deficiency Promotes Motor Deficits of a C9ALS/FTD Mouse Model in a Dose-Dependent Manner. Acta Neuropathol. Commun..

[60] Zhu Q., Jiang J., Gendron T.F., McAlonis-Downes M., Jiang L., Taylor A., Diaz Garcia S., Ghosh Dastidar S., Rodriguez M.J., King P. (2020). Reduced C9ORF72 Function Exacerbates Gain of Toxicity from ALS/FTD-Causing Repeat Expansion in C9orf72. Nat. Neurosci..

[61] Hu J., Rigo F., Prakash T.P., Corey D.R. (2017). Recognition of C9orf72 Mutant RNA by Single-Stranded Silencing RNAs. Nucleic Acid Ther..

[62] Kramer N.J., Carlomagno Y., Zhang Y., Almeida S., Cook C.N., Gendron T.F., Prudencio M., Van Blitterswijk M., Belzil V., Couthouis J. (2016). Spt4 Selectively Regulates the Expression of C9orf72 Sense and Antisense Mutant Transcripts. Science.

[63] Martier R., Liefhebber J.M., García-Osta A., Miniarikova J., Cuadrado-Tejedor M., Espelosin M., Ursua S., Petry H., van Deventer S.J., Evers M.M. (2019). Targeting RNA-Mediated Toxicity in C9orf72 ALS and/or FTD by RNAi-Based Gene Therapy. Mol. Ther. Nucleic Acids.

[64] Mueller C., Berry J.D., McKenna-Yasek D.M., Gernoux G., Owegi M.A., Pothier L.M., Douthwright C.L., Gelevski D., Luppino S.D., Blackwood M. (2020). Suppression with Adeno-Associated Virus and MicroRNA in Familial ALS. N. Engl. J. Med..

[65] Pinto B.S., Saxena T., Oliveira R., Méndez-Gómez H.R., Cleary J.D., Denes L.T., McConnell O., Arboleda J., Xia G., Swanson M.S. (2017). Impeding Transcription of Expanded Microsatellite Repeats by Deactivated Cas9. Mol. Cell.

[66] Batra R., Nelles D.A., Pirie E., Corbett K.D., Swanson M.S., Yeo G.W. (2017). Elimination of Toxic Microsatellite Repeat Expansion Article Elimination of Toxic Microsatellite Repeat Expansion RNA by RNA-Targeting Cas9. Cell.

[67] Zamiri B., Reddy K., Macgregor R.B., Pearson C.E. (2014). TMPyP4 Porphyrin Distorts RNA G-Quadruplex Structures of the Disease-Associated r (GGGGCC) n Repeat of the C9orf72 Gene and Blocks Interaction of RNA-Binding Proteins *. J. Biol. Chem..

[68] Simone R., Balendra R., Moens T.G., Preza E., Wilson K.M., Heslegrave A., Woodling N.S., Niccoli T., Gilbert-Jaramillo J., Abdelkarim S. (2018). G-quadruplex-binding Small Molecules Ameliorate C9orf72 FTD/ALS Pathology in Vitro and in Vivo. EMBO Mol. Med..

[69] Zhang Q., An Y., Chen Z.S., Koon A.C., Lau K.F., Ngo J.C.K., Chan H.Y.E. (2019). A Peptidylic Inhibitor for Neutralizing r (GGGGCC) Exp-Associated Neurodegeneration in C9ALS-FTD. Mol. Ther. Nucleic Acids.

[70] Jiang J., Ravits J. (2019). Pathogenic Mechanisms and Therapy Development for C9orf72 Amyotrophic Lateral Sclerosis/Frontotemporal Dementia. Neurotherapeutics.

[71] Zhou Q., Lehmer C., Michaelsen M., Mori K., Alterauge D., Baumjohann D., Schludi M.H., Greiling J., Farny D., Flatley A. (2017). Antibodies Inhibit Transmission and Aggregation of C9orf72 Poly-GA Dipeptide Repeat Proteins. EMBO J..

[72] Nguyen L., Montrasio F., Pattamatta A., Tusi S.K., Bardhi O., Meyer K.D., Hayes L., Nakamura K., Banez-Coronel M., Coyne A. (2020). Antibody Therapy Targeting RAN Proteins Rescues C9 ALS/FTD Phenotypes in C9orf72 Mouse Model. Neuron.

[73] Cristofani R., Crippa V., Vezzoli G., Rusmini P., Galbiati M., Cicardi M.E., Meroni M., Ferrari V., Tedesco B., Piccolella M. (2018). The Small Heat Shock Protein B8 (HSPB8) Efficiently Removes Aggregating Species of Dipeptides Produced in C9ORF72-Related Neurodegenerative Diseases. Cell Stress Chaperones.

[74] Moseley M.L., Zu T., Ikeda Y., Gao W., Mosemiller A.K., Daughters R.S., Chen G., Weatherspoon M.R., Clark H.B., Ebner T.J. (2006). Bidirectional Expression of CUG and CAG Expansion Transcripts and Intranuclear Polyglutamine Inclusions in Spinocerebellar Ataxia Type 8. Nat. Genet..

[75] Cho D.H., Thienes C.P., Mahoney S.E., Analau E., Filippova G.N., Tapscott S.J. (2005). Antisense Transcription and Heterochromatin at the DM1 CTG Repeats Are Constrained by CTCF. Mol. Cell.

[76] Zu T., Guo S., Bardhi O., Ryskamp D.A., Li J., Khoramian S. (2020). Metformin Inhibits RAN Translation through PKR Pathway and Mitigates Disease in C9orf72 ALS/FTD Mice. Proc. Natl. Acad. Sci. USA.

[77] Hautbergue G.M., Castelli L.M., Ferraiuolo L., Sanchez-martinez A., Cooper-knock J., Higginbottom A., Lin Y., Bauer C.S., Dodd J.E., Myszczynska M.A. (2017). SRSF1-Dependent Nuclear Export Inhibition of C9ORF72 Repeat Transcripts Prevents Neurodegeneration and Associated Motor Deficits. Nat. Commun..

[78] Becker L.A., Huang B., Bieri G., Ma R., Knowles D.A., Jafar-Nejad P., Messing J., Kim H.J., Soriano A., Auburger G. (2017). Therapeutic Reduction of Ataxin-2 Extends Lifespan and Reduces Pathology in TDP-43 Mice. Nature.

[79] Xi Z., Zinman L., Moreno D., Schymick J., Liang Y., Sato C., Zheng Y., Ghani M., Dib S., Keith J. (2013). Hypermethylation of the CpG Island near the G4C2 Repeat in ALS with a C9orf72 Expansion. Am. J. Hum. Genet..

[80] Bravo-hernandez M., Tadokoro T., Navarro M.R., Platoshyn O., Kobayashi Y., Marsala S., Miyanohara A., Juhas S., Juhasova J., Skalnikova H. (2020). Spinal Subpial Delivery of AAV9 Enables Widespread Gene Silencing and Blocks Motoneuron Degeneration in ALS. Nat. Med..

